# Histomorphometric Evaluation of New Bone Formation, Dimensional Changes, and Residual Particles in Alveolar Ridge Preservation Techniques Using InterOss® Anorganic Cancellous Bone Graft: A Longitudinal Study

**DOI:** 10.1155/2024/3263011

**Published:** 2024-05-15

**Authors:** Melanie Karoly Elizalde-Mota, Cindy Hernández-Romero, Sergio Sanchez-Sosa, Valeria Magali Rocha–Rocha, Maritza Espinosa-Arreola

**Affiliations:** ^1^Popular Autonomous University of the State of Puebla, Puebla, Mexico; ^2^Surgical and Molecular Pathology, Angeles Hospital, LABOPAT, Puebla, Mexico; ^3^Interdisciplinary Postgraduate Center, Popular Autonomous University of the State of Puebla, Puebla, Mexico; ^4^Health Sciences, Popular Autonomous University of the State of Puebla, Puebla, Mexico

## Abstract

**Materials and Methods:**

We worked on 14 single-root or premolar teeth with fused roots with an indication for the preservation of the alveolar ridge and the subsequent placement of a dental implant. The techniques performed in the study were the Bartee technique for the preservation of the alveolar crest in seven teeth that presented a good amount of the remaining bone tissue (minimum 4 mm in the apical–coronal direction) or that had a buccal or lingual/palatine wall defect (group A) and the Bio-Col alveolar preservation technique (group B), in seven teeth that presented an intact alveolus (four remaining walls). Xenograft was used in both groups. *Results:* Morphometric evaluation of group A (Bartee technique) and group B (Bio-Col technique) showed 11.48% and 13.24% of reabsorption in the vertical direction (*p*=0.482) and 21.95% and 20.55% in the horizontal direction, respectively (*p*=0.949). At 6 months of healing, the histomorphometric evaluation showed 31.10% new bone formation and 28.09% residual particles in group A, 13.24% new bone formation (*p*=0.744), and 20.55% residual particles for group B (*p*=0.302). There were no statistically significant differences in dimensional changes between both groups.

**Conclusions:**

The Bartee and Bio-Col alveolar ridge preservation technique combined with a xenograft provides dimensional stability, counteracting the physiological resorption process and ensuring the stability of the surrounding tissues. Therefore, both techniques represent a predictable option for dental implant placement at 6 months of healing.

## 1. Background

The indication for tooth extraction happens when it cannot be restored or kept in acceptable long-term conditions regarding health, function, and esthetics [1].

Consequently, the absence of a single or multiple teeth triggers a cascade of irreversible biological events, which generally results in significant local anatomical changes in the height and width of the residual alveolar ridge. The amount of bone resorption in width has been considered the most affected, mainly in the vestibular wall of the socket, while the changes produced in the other walls of the socket are minor [2, 3].

The resorption of extraction sites results in narrower and thinner ridges with reduced height and a lingual/palatal displacement of the axial longitudinal axis of the socket [4].

In the same way, several studies support the unfavorable results caused by the progressive resorption process in the residual alveolar ridge, which is noticeable only in the first 3, 6, and 12 months of healing, impairing the placement of conventional or implant-supported dentures [2, 4, 5].

The bone resorption process triggers a gradual bone remodeling, that is, a change in size and shape, and a remodeling of the existing bone tissue after the tooth extraction, reporting up to 40% in height and a 60% width only in the first 6 months of healing [6].

Surgical trauma caused by tooth extraction can induce microtrauma to the surrounding bone tissue, accelerating the bone remodeling process in the residual alveolar ridge [7].

Therefore, bone atrophy is a common clinical issue, making it difficult to place a dental implant; concerning the above, over the years, surgical techniques have been developed to treat this consequence, among which include guided bone regeneration (GBR), block grafts, sinus floor elevation, bone distractions, alveolar nerve transposition, mediodistally tilted implants, or the use of pterygoid, zygomatic implants. The short implants seem to be a simplified, minimally invasive alternative. Nevertheless, the angulated abutments introduced the possibility of tilting the distal implants to avoid the anatomic boundaries (alveolar nerve and maxillary sinus). However, these surgical procedures can result in prolonged treatment times, high costs, and morbidity for the patient [8, 9].

In the last two decades, various surgical procedures have emerged; the purpose of such procedures is to maintain an ideal alveolar ridge that allows for maintaining an adequate esthetic profile in the anterior area and preventing the collapse of the residual alveolar ridge; guided bone regeneration simultaneously with tooth extraction has the advantage of preserving bone volume by counteracting the bone resorption process, thus preserving the adequate dimensions of the bone tissue due to the facilitate the correct placement of a dental implant [10, 11].

Alveolar ridge preservation arose through the “grafted socket,” which emerged in the mid-1980s as an alternative to root immersion carried out at that time to preserve the bone contour. Its use became popular with the objective of “filling” the postextraction socket with a biomaterial, which sought to preserve the dimensions of the socket, which would facilitate the placement of endosseous implants [12].

On the other hand, according to the literature, biological mediators improve the alveolar healing process. Platelets have many functions beyond the hemostatic one. Activated platelets released growth factors and cytokines such as fibrinogen, primary fibroblast growth factor, fibronectin, angiopoietin-2, insulin-like growth factor-I, platelet-derived growth factor, transforming growth factor *β*1 (TGF *β*1), and vascular endothelial growth factor which play an essential role in soft and hard tissue healing [13].

Human mesenchymal stem cells are the foundation of any tissue engineering approach aiming at the regeneration of mineralized tissues; historically, one of the first sites to derive mesenchymal stem cells is the bone marrow; however, the procedure is difficult to access and often painful.

Recent studies report the extraction of mesenchymal stem cells from isolates of the dental pulp of the third molar and buccal fat pads, considered an essential reservoir of said cells, thus being an alternative for regenerative procedures [14].

There are multiple techniques for alveolar ridge preservation. The most popular are Bartee and the Bio-Col technique. The Bartee technique is assisted by a nonresorbable dense polytetrafluoroethylene membrane (PTFE-d), specifically designed for use in regenerative procedures, which do not require a primary closure; it avoids the migration of particles from the bone graft at the same time as previous migration of the soft and epithelial tissue during the healing process, being an essential requirement in guided bone regeneration procedures; the blocking of epithelial migration in bone defects results in a potentiated regeneration of bone tissue, through the selective cellular repopulation of the wound by osteoprogenitor cells, thanks to the local concentration of biological growth factors [12]. Healing kinetics at the extraction sites look similar, as there is a tendency for soft tissue invagination and fibrous tissue formation in the coronal third of the socket that does not require primary closure; therefore, it seems that facilitates the preservation of the keratinized gingiva and gingival architecture, as well as an improvement in the color of the healing provided by a highly vascularized bone tissue free of fibrosis or chronic inflammation at 6 months of healing in alveolar ridge preservation [15].

The Bio-Col technique uses a resorbable collagen dressing that promotes guided bone regeneration for at least 30 days, provides encouraging results in maintaining soft tissues, and minimizes resorption in the socket. It is as effective as other membranes of guided tissue regeneration in the inhibition of epithelial migration and in promoting a new connective tissue attachment [16]. Likewise, resorbable collagen dressing is preferable due to its physiological absorption process and high biocompatibility with oral tissues [17]. In the same way, collagen is a hemostatic agent and can stimulate platelet aggregation and improve fibrin binding, leading to initial clot formation, stability, and maturation. Collagen is chemotactic for fibroblasts in vitro studies; in general terms, this property could improve cell migration and thus promote the primary closure of the wound, which is considered essential to achieve bone growth [18].

There are reports describing that after dental extraction without reflecting a flap, in a short healing period (3 weeks), the xenograft and allograft present less bone resorption in the alveolar ridge than alloplastic or physiological scarring [19].

The management of soft tissues during tooth extraction is of utmost importance to maintain an ideal contour for the peri-implant tissue, as well as the esthetic and functional requirements; there are adverse effects when reflecting a flap during tooth extraction, they have mentioned an impact on bone remodeling due to periosteal damage, decreased vascular supply, and postoperative inflammation [20].

Some biomaterials used in bone substitutes are autografts, obtained from the same patient; allografts, from the same species, that is, from another human being; xenografts of animal origin (coral, plants, or animals); and alloplastics graft of synthetic origin. Their permanency ensures they maintain space and act as a scaffold for new bone growth; however, no grafting material can prevent bone resorption completely [21–23].

Autografts have the potential to form new bone through osteogenesis, osteoinduction, and osteoconduction and have always served as a gold standard for regeneration. However, autogenous bone grafts have several disadvantages, such as limited material, morbidity at the donor site, bone quality, and unpredictable and postoperative discomfort [24].

Alloplastic grafts are synthetic bone substitutes that act as biological fillers; they are osteoconductive bone substitutes, and bioactive glass has been used in alveolar ridge preservation. This graft material can adhere to normal bone, help its remodeling, and enable hemostasis, which presents a clinical advantage in the quality of the regenerated bone after 6 months [25].

Freeze/dried bone allograft (FDBA) and demineralized freeze-dried cortical bone allograft (DFDBA) have osteoconductive capabilities and fast resorption, with bone ingrowth. DFDBA also showed more vital bone and less residual grafting material than FDBA when placed in alveolar ridge preservation 19 weeks after extraction [26].

As reported in the literature, a few studies have directly compared different grafting materials in alveolar ridge preservation, with allograft and xenograft being the most studied [26, 27].

Deproteinized bovine bone mineral (DBMM) is a xenograft reduced to pores of different dimensions (0.25–2 mm) and deprived of all its organic components by various means to avoid an immune response, leaving a bone mineral matrix of inorganic crystalline hydroxyapatite, which is biocompatible and similar both physically and chemically to human bone. It provides an osteoconductive activity and has a slow resorption rate, contributing to maintaining the volume. It has been considered one of the most used biomaterials for alveolar ridge preservation; various preclinical and clinical studies support biocompatibility and integration with newly formed bone tissue [27–29].

The objective of the present study was to compare through histological, histomorphometric, and morphometric evaluation of new bone formation in the alveolar ridge preservation between the Bartee and Bio-col technique with the use of xenograft at 6 months of healing, based on the literature that supports the xenograft's maturation at this time of healing, finding a mature bone matrix and encapsulated bone particles surrounded by vital bone tissue [1, 11, 14, 27, 30–32]. We compared the dimensional changes by cone-beam computed tomography (CBCT) evaluation in pre- and postpreserved sites.

## 2. Materials and Methods

The Research Ethics Committee of the Department of Health Sciences from Popular Autonomous University of the State of Puebla (CONBIETICA21CEI00620131021) approved this study. There were four patients in this study; the patients involved had an age range between 59 and 63 years, an average of 61.25 years. They required the extraction of single-rooted teeth or fused premolar roots and the subsequent placement of a dental implant.

All patients were systemically healthy, and study inclusion was 14 teeth (patient 1, six teeth; patient 2, five teeth; patient 3, one tooth; and patient 4, two teeth) of single-rooted or fused premolar roots located in mandibular or maxillary which were indicated for extraction (the leading causes of extraction were advanced carious lesions, endodontic treatment failure, and root fracture) and subsequent dental implant placement based on the inclusion criteria for each technique.

The inclusion criteria for the Bartee socket preservation technique are as follows: seven uniradicular teeth or premolars with fused roots with a good amount of remaining bone tissue (minimum 4 mm in the apical–coronal direction) and presence of sufficient residual alveolar bone volume to achieve primary implant stability, which present a defect in the vestibular or lingual/palatal wall.

The inclusion criteria for the Bio-Col alveolar preservation technique are as follows: seven uniradicular teeth or premolars with fused roots with a good amount of remaining bone tissue (minimum 4 mm in the apical–coronal direction) and presence of sufficient residual alveolar bone volume to achieve primary implant stability, which present an intact alveolus (four remaining walls).

The exclusion criteria for both techniques included patients with ASA (American Society of Anesthesiologists) III, IV, and V condition, addiction to alcohol or drugs, smokers >10 cigarettes per day, psychiatric problems, an active periodontal disease associated with poor hygiene and without oral motivation, acute infection (abscess) or presence of purulent discharge at or near the site to be preserved, traumatic tooth extraction injuring surrounding soft and hard tissue, malignant disease, and diseases treated with chemotherapy or radiotherapy.

Phase I periodontal treatment was performed in all patients following the European Federation of Periodontology guidelines, individual advice on good oral hygiene for optimal response to treatment, and long-term plaque control. Each patient had professional mechanical plaque removal and individual advice for supportive periodontal care. Preoperative clinical examinations were requested (six-element blood chemistry, hematic biometry, prothrombin time, and partial thromboplastin time).

### 2.1. Surgical Technique

One periodontist performed all surgical procedures in this study, using xenograft in both groups, performing extraoral asepsis and antisepsis with povidone–iodine 10% and chlorhexidine rinses before anesthesia with articaine 4%/epinephrine 1:100,000 and atraumatic tooth extraction. After washing and curetting the socket(s), we performed the alveolar ridge preservation technique indicated in each case.

**Figure 1:**
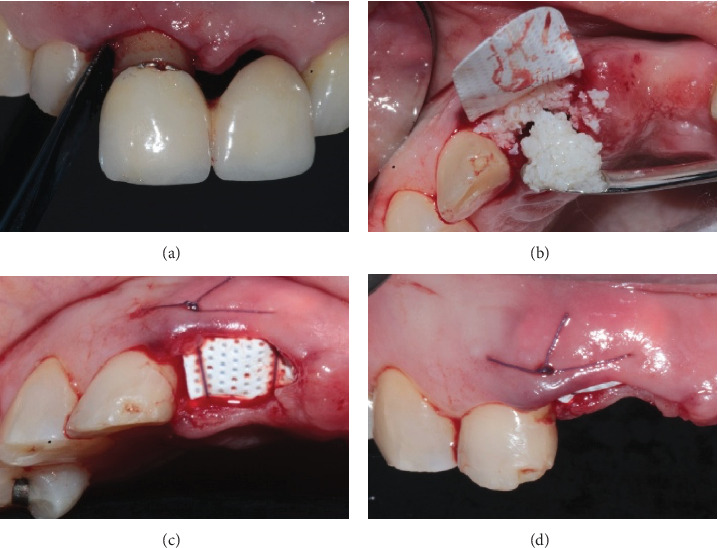
(a) Atraumatic extraction. (b) Nonresorbable membrane fitting. (c) Bartee technical suture. (d) Bartee technical adaptation and vestibular stability of the nonresorbable membrane.

The alveolar ridge preservation Bartee technique (group A) was performed in seven teeth with a good amount of remaining bone tissue (minimum 4 mm in apical–coronal direction) or a vestibular or lingual/palatal wall defect. The xenograft was placed in the entire socket assisted by a nonresorbable dense polytetrafluoroethylene membrane (Cytoplast™ Regentex TXT-200 singles, Osteogenics Biomedical Inc., Lubbock, TX), removing it at 21 days of healing, and avoiding excessive compaction of the graft. Interrupted sutures were used in the interdental space, papillae, and single or horizontal mattress sutures through the socket opening (4–0 polyglycolic (Vicryl, Ethicon Inc., Somerville, NJ); in the case of needing to place a provisional, it was placed after the surgical procedure (Figure 1).

**Figure 2:**
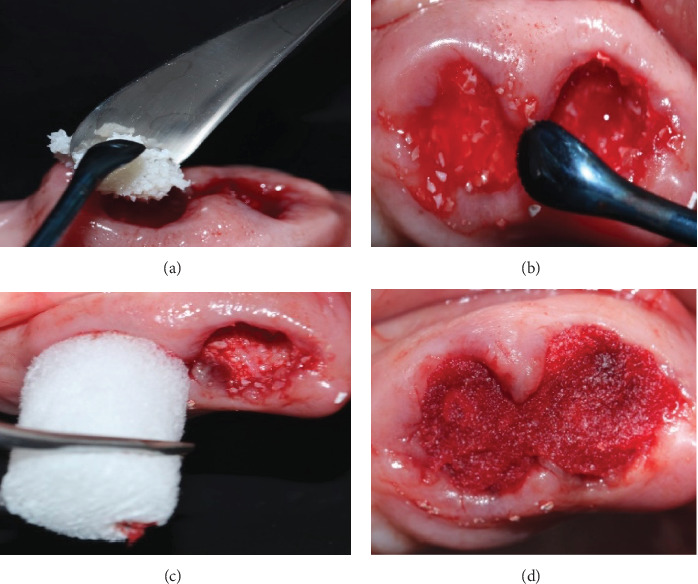
(a) Atraumatic extraction and xenograft placement. (b) Xenograft was placed in three-fourths of the socket. (c) The last one-fourth of the socket was occupied by collagen resorbable dressing. (d) Vascular hydration of the collagen dressing in the last one-fourth of the socket.

The Bio-Col alveolar preservation technique (group B) was performed in seven teeth, which presented an intact socket (four remaining walls), using xenograft in three-fourths of the socket and the last one-fourth resorbable collagen dressing (CollaPlug® Zimmer biomet). It was closed with a crossed suture (vicryl 4.0® polyglactin 910 Johnson & Johnson Medical Devices & Diagnostics Group), and tissue adhesive stabilized the socket and the suture (Periacryl 90-HV® GluStitch Inc.) (Figure 2).

The postoperative care and the prescription provided to each patient were antibiotic therapy (amoxicillin 500 mg one tablet every 8 hr for 7 days) and pain and edema controlled with ibuprofen (400 mg one tablet every 6 hr for 2 days), no use of dental prostheses for 4 weeks, and to rinse twice daily with 0.12% chlorhexidine digluconate for the first 2 weeks and to use modified oral hygiene procedures in the treated area for the first 4 postoperative weeks. Patients were strictly advised not to smoke during the healing period and not to rub the surgical area for 2 weeks. The follow-up appointment was 7 days after surgery, suture removal at least 14 days later, and regular oral hygiene practices 4 weeks later. All patients returned for a professional tooth cleaning after 1, 2, 3, and 4 weeks and after 3 and 6 months.

### 2.2. Histological and Histomorphometric Evaluation

**Figure 3:**
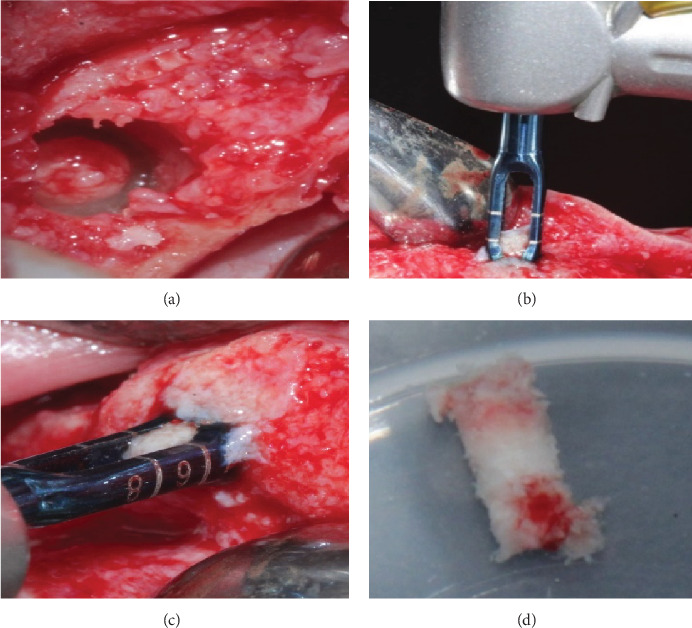
(a) Six-month healing Bio-Col technique. (b) Sampling at 6 months of healing with trephine of 2 mm diameter by 4 mm length Bio-Col technique. (c) Sampling at 6 months of healing with trephine of 2 mm diameter by 4 mm length Bartee technique. (d) Fixation of biopsy in 10% formalin.

Six months after the alveolar ridge preservation, we collected bone biopsies using a trephine bur of 2 mm diameter by 4 mm long at the implant placement site. We fixed them in 10% neutral formalin for 6–24 hr after decalcifying using Osteosoft^©^ for 24 hr (Osteosoft (Sigma–Aldrich), its formulation based on ethylenediaminetetraacetic acid) (Figure 3).

The samples were placed in an automatic tissue processor for paraffin and tissue block inclusion, making 4 *µ*m sections for hematoxylin and eosin (H&E), Masson, and Reticulum stains from each biopsy.

Immunohistochemistry was performed using the Ventana immunostainer (BenchMark Sistem Roche TM) and a monoclonal CD34 antibody (Biocare 1:100).

The slides were observed under a microscope (trinocular fluorescence microscope BX-41, Olympus®) with magnifications of 10x, 20x, and 40x; percentages of total bone volume residual particles were obtained using the software (Jenoptik Software Gryphax®) for image evaluation.

**Figure 4:**
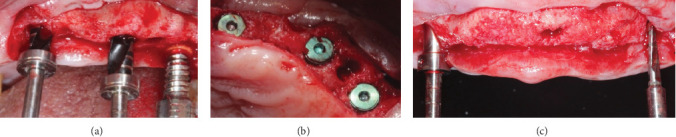
(a) Drilling protocol, (b) implant placement, and (c) parallelism and drilling protocol (JDEvolution® Plus).

We place dental implants (JDEvolution® Plus with sand-blasted large grit acid-etched surface) in the 14 preserved sites, based on an individual rehabilitation plan for each patient, with diameters 3.7, 4.2 mm, and length 10, 11.5, and 13 mm. The sample collection area was used as the start of the drilling protocol (Figure 4).

### 2.3. Morphometric Evaluation by Cone Beam Computed Tomography

**Figure 5:**
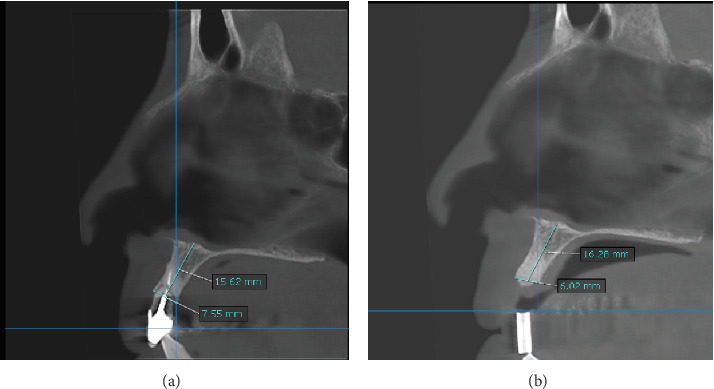
(a) Baseline measure CBCT of the upper central incisor (Bartee technique). (b) Measure of 6 months of healing.

**Figure 6:**
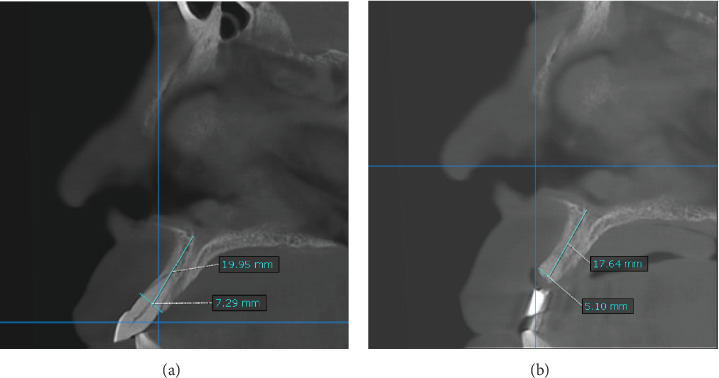
(a) Baseline measure CBCT of the lateral incisor (Bio-Col technique). (b) Measure of 6 months of healing.

In morphometric analysis, we measured the height and width of the socket with two CBCTs; the first CBCT before performing alveolar ridge preservation (baseline measurements), evaluating the presence or absence of the buccal plate, periapical lesion, root fracture, and root length; the second CBCT was taken at 6 months of healing; a baseline measurement was recorded at 6 months, coinciding in the cutting plane to evaluate the dimensional change of the socket (Figures 5 and 6).

### 2.4. Statistical Analysis

The data were analyzed using the IBM SPSS Statistics database for Windows, version 25 (IBM Corp., Armonk, NY, USA).

We calculated frequencies, percentages, mean, and standard deviation. We used the Wilcoxon signed-rank test for related samples and the Mann–Whitney *U* test for two unrelated samples. A *p* value <0.05 was considered statistically significant.

## 3. Results

The general characteristics of the patients, alveolar ridge preservation site, and implant placement site included in the present study are described in Table 1.

### 3.1. Histomorphometric Evaluation

When performing the morphometric analysis by CBCT, the percentage of resorption obtained in group A and group B in the vertical direction was 11.48% and 13.24% of resorption (*p*=0.482) and in the horizontal direction 21.95% and 20.55%, respectively (*p*=0.949), thus demonstrating that there is no statistically significant difference (*p* > 0.05) between both techniques (Table 2).

As a result, in the histomorphometric evaluation, the percentage of new bone tissue formed for group A was 31.10%. In comparison, for group B, it was 30.95% (*p*=0.744), referring to the percentage of residual particles represented 28.09% for group A and group B with 26.83% (*p*=0.302) at 6 months of healing (Table 3), so there is no statistically significant difference in terms of dimensional changes, new bone formation, and residual particles between both techniques (*p*  > 0.05).

### 3.2. Histological and Immunohistochemical Evaluation

**Figure 7:**
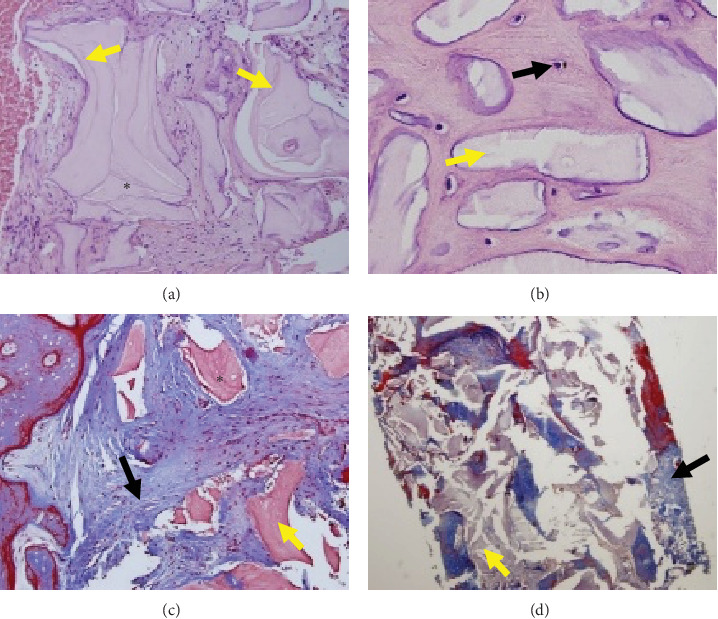
Healing at 6 months, (a) 10x magnification, H&E stain, presence of trabeculae (yellow arrows) of variable thickness, fibrous stroma, (*⁣*^*∗*^) intertrabecular lines of fusion between two residual particles. (b) 40x magnification, H&E stain, spicules, and own trabeculated bone with the presence of osteocytes (black arrow) as well as residual particles (yellow arrow) eosinophilic character with less density and lacking osteocytes. (c) 10x magnification, Masson's trichrome stain, grafted bone immersed in a collagenized fibrous matrix, presence of blood vessel (yellow arrow), and own reactive bone (black arrow). (d) 4x magnification, panoramic view (visualization of both components), Masson's trichrome stain, presence of residual particles (yellow arrow), and own reactive bone (black arrow).

The histological evaluation of the 14 biopsies consisted of newly formed bone and residual particles, in intimate contact with the newly formed bone; no necrosis or foreign body reactions were detected. The residual particle was present in 6-month sections by H&E, Masson's stain, and cross-link staining. The outlines of the residual bone substitute (xenograft) particles were detectable due to the change in density (Figure 7).

The newly formed bone consisted of woven and laminar bones and appeared as vital bone tissue containing osteoblasts, an osteoid covering the rim.

**Figure 8:**
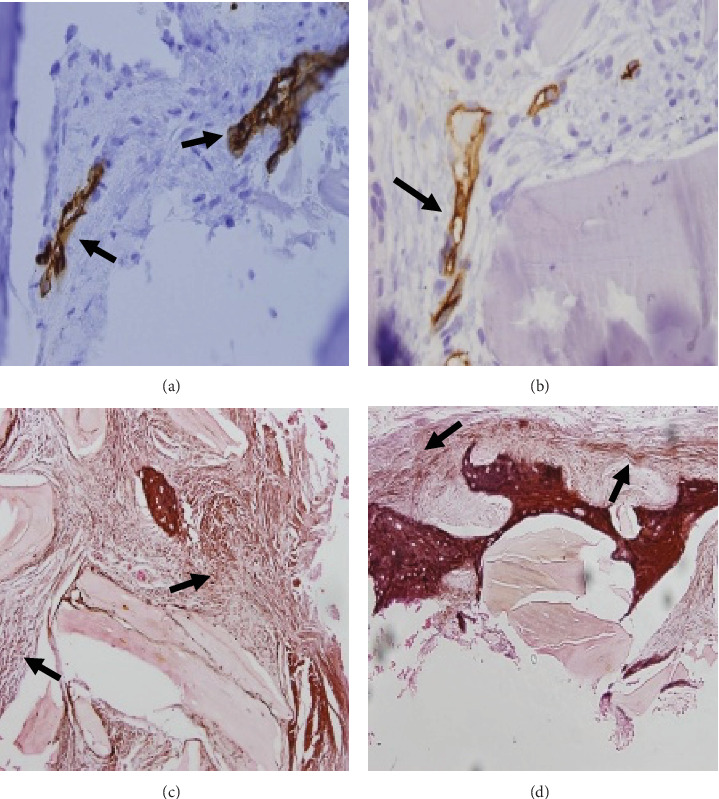
Healing at 6 months, (a) 20x magnification, immunohistochemistry CD34 monoclonal antibody, and endothelial cells lining intertrabecular stromal vessels (black arrows). (b) 20x magnification, immunohistochemistry CD34 monoclonal antibody, endothelial cell lining intertrabecular stromal vessels (black arrows), and vessel proliferation. (c) 10x magnification, reticular stain, and reticular fiber condensation (black arrows). (d) 20x magnification, reticulum stain, and reticular fiber condensation (black arrows).

Histological findings did not show inflammatory cells in the tissue in close contact with the bone substitute particles (Figure 8).

**Figure 9:**
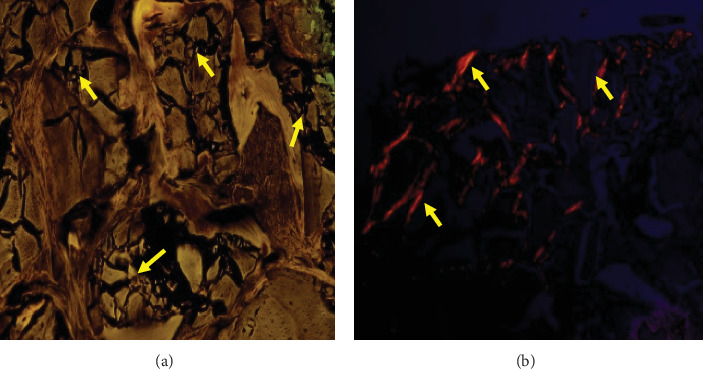
Healing at 6 months, (a) 10x magnificatiification, immunofluorescence, Texas red filter, and collagen autofluorescence (yellow arrows). On, polarized light, allows observing fragmented residual particles (yellow arrows), puzzle appearance. (b) 10x magnification.

Fragmentation zones are observed in the residual particles, simulating a puzzle in polarized light analysis. In the same way, there was a change in density in the analysis with immunofluorescence that allows differentiation between the newly formed bone and the residual particles due to the lack of collagen in the latter (Figure 9).

**Figure 10:**
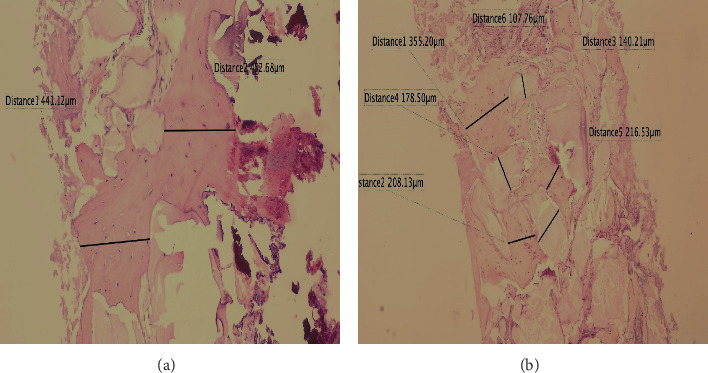
Healing at 6 months, (a) 10x magnification, H&E stain, and bone trabeculae were measured in *µ*m. (a) Panoramic view 4x, H&E stain, bone trabeculae, and residual particles were measured in micrometers.

The bone trabeculae present and the residual particles in *µ*m were measured in percentage using the software (Jenoptik Software Gryphax®) for image evaluation; these measurements were represented in percentage in the statistical analysis (Figure 10).

## 4. Discussion

The present study showed that using a xenograft combined with the Bartee and Bio-Col alveolar ridge preservation technique could counter the physiological resorption process after tooth extraction. There are various biocompatible materials to treat bone atrophy secondary by tooth extraction. The success rate for implants placed in regenerated bone is comparable to those placed in native bone. The alveolar ridge dimensions are so critical that alveolar preservation after a tooth extraction is essential to maintain the vertical and horizontal dimensions of the alveolar ridge [12].

Fischer et al. [31] reported a 14% greater loss in soft and hard tissue remodeling after tooth extraction by lifting a mucoperiosteal flap in a 3-month healing period. Concerning the above, the extractions were without raising a mucoperiosteal flap in both groups or causing damage to the surrounding tissue, which favors the healing process.

A large number of articles in the literature supports and studies the bone resorption produced by tooth extraction; a higher percentage of bone resorption is expected in the horizontal direction compared to the loss in the vertical direction [1, 2, 33–35]; this result is corroborated in the present study, obtaining a more significant loss in the horizontal direction in both techniques.

In a randomized controlled clinical trial, dimensional evaluation was performed in different techniques of alveolar preservation in the posterior sector in four study groups using DBBM. The evaluation, after 6 months of healing, observed that there was no statistically significant difference in dimensional changes (<1 mm) [35]. In the present study, we found no statistically significant difference in terms of dimensional changes in the Bartee and Bio-Col alveolar preservation technique at 6 months of healing using xenograft, providing dimensional stability for the future placement of a dental implant, thus reducing the need for a second regenerative surgical procedure.

Tan et al. [36] reported the percentage of dimensional changes in a systematic review after tooth extraction without intervening in the healing process ranging between 11% and 22% and 29% to 63% bone resorption in the vertical and horizontal direction respectively after 6–7 months of healing [36]. On the other hand, when using xenograft in alveolar preservation using the Bartee or Bio-col technique, the dimensional stability of the alveolus was observed in the present study, obtaining a percentage of resorption (11.48%–13.24%) in the vertical direction and (20.55%–21.95%) in horizontal direction. Counteracting the physiological resorption process triggered by tooth extraction.

The clinical study by Schropp et al. [37] reported that the volume reduction in the horizontal direction in the alveolar crest after tooth extraction was, on average, 5–7 mm only in the first 12 months. They also reported that these values are equivalent to 50% of alveolar bone in the presence of the tooth [32]. The results coincide with those reported in the present study, in which a more significant loss is observed in the horizontal direction in group A at 21.95% and group B at 20.55%.

On the other hand, in a systematic review of 32 studies, 1,354 alveolar ridge preservations refer to the volume obtained in the socket healing using biomaterials such as xenograft, allograft, and alloplastic; it was reported that the xenograft and allograft bone graft showed less dimensional loss than the nongrafted socket or grafted with alloplastic, as well as the use of xenograft to perform alveolar ridge preservation which was the most reported and studied (21 of the 32 studies analyzed) with predictable short-term results, in the same way its combination with a membrane as a barrier or collagen resorbable for the correct sealing and isolation of the socket, so the use of xenograft is suggested to perform alveolar ridge preservation [19]; in this sense by using a dense absorbable polytetrafluoroethylene membrane or a collagen dressing in conjunction with a xenograft was observed in both cases by morphometric analysis with CBCT an adequate bone volume at 6 months of healing, reporting that the dimensions of the residual alveolar ridge, bone quality, and quantity of bone tissue for the future placement of a dental implant, were notably higher with the use of a xenograft, coinciding with what was reported in the present study.

A recent systematic review and meta-analysis evaluate and compare the effects of different graft materials (xenograft, allograft, autograft, and control group) used in alveolar ridge preservation on dimensional complex tissue changes of the alveolar ridge, assessed using cone-beam computed tomography scans. The less vertical and horizontal bone reduction was observed with xenogenic graft material as opposed to allogenic graft; however, the loss of alveolar ridge dimensions could not be prevented entirely by any graft material. Moreover, there is currently insufficient evidence to compare the effectiveness of autogenic graft materials in alveolar ridge preservation techniques based on radiological assessments using CBCT scans [14, 38]. Finding similar cone-beam computed tomography scans results through the use of alveolar preservation xenograft in combination with a nonresorbable membrane and collagen dressing.

Based on the study by Barone et al. [39], when using xenograft in alveolar ridge preservation versus tooth extraction at 7 months of healing, obtaining by histomorphometric analysis 25.7%–9.5% of trabecular bone in the tooth extraction group, the connective tissue constituted 59.1%–10.4% of the area total studied. In contrast, the amount of trabecular bone for the alveolar preservation group was 35.5%–10.4%; the connective tissue was from 36.6% to 12.6%, and the residual particles observed were from 29.2% to 10.1% [39]. With the Bartee technique, we observed 31.10% of trabecular bone formed, while the number of residual particles represented 28.09%; on the other hand, in the Bio-Col group, the trabecular bone formed constituted 30.95%, as well as residual particles of 26.83%. In both techniques, the residual particles were in close contact with the bone tissue.

The present outcomes agree with findings from clinical studies indicating using bovine-derived xenograft with 10% collagen and collagen membrane in alveolar ridge preservation at 6-month healing [32]. Modifying the marginal ridge resorption after tooth extraction could more successfully preserve the complex tissue dimension than allowing spontaneous healing by Iorio-Siciliano et al. [32]

Another study compared allograft versus xenograft used in alveolar ridge preservation assisted by a resorbable collagen membrane, reporting no statistically significant difference between both groups at 6 months of healing [27]. In particular, when using a resorbable collagen dressing in conjunction with a xenograft, it was demonstrated that there is dimensional stability of the residual alveolar ridge in single-rooted sites employing CBCT at 6 months of healing, coinciding with that reported by Méndez et al. [28].

Vaia et al. [40] report a clinical and histomorphometric study about the use of deproteinized bovine bone mineral and xenogeneic collagen matrix in the preservation of the alveolar ridge at 12 months, finding an average of 29.52% ± 6.09% of samples, and they were embedded in newly formed bone, representing an average of 27.72% ± 5.64% of the samples. It is considered a predictable technique that provides favorable conditions for implant placement in the anterior maxilla [32]. Finding similar results in the present study regarding the percentage of new bone was 31.10% for group A, and for group B, it was 30.95%

A recent systematic review reported that autogenous particulate dentin is a good option as a graft material in alveolar ridge preservation procedures due to its osteoconductive and osteoinductive properties [40].

In the literature, there is evidence that platelet concentrates may be advantageously used in postextraction sites, mainly to improve soft tissue healing and reduce postoperative symptoms, enhancing the alveolar healing process using biological mediators [13, 14]. After 6 months of healing, a histological study reported the presence of inflammatory cells surrounding the bone tissue; therefore, using platelet concentrate to reduce the inflammatory process in conjunction with alveolar ridge preservation seems to be a treatment option.

A histological evaluation performed on alveolar bone sites using a nonresorbable membrane (d-PTFE) at 12 months of healing indicated that the newly formed tissue was mainly trabecular bone with areas of bone marrow with lymphocytes and rarely granulocytes, osteocytes, and osteoblasts. These findings indicate that active bone formation was present, similar to bone found in healed extraction sites where bone graft placement was not performed. These histological results agree with those reported in the present study, where trabecular bone and bone marrow areas are reported 6 months after healing [41].

## 5. Conclusions

The Bartee and Bio-Col alveolar ridge preservation technique, in combination with a xenograft, provides favorable results after 6 months of healing, providing a more favorable environment for the peri-implant tissue, thus increasing the treatment options and combination of these, reporting maintenance of the residual alveolar ridge volume, formation of vital bone, and residual particles in close contact with the new bone formed in both techniques.

The morphometric and histomorphometric analysis of the results did not find a significant difference between both techniques, a lower percentage in height and width of the socket, residual particles, or a more significant formation of new bone that suggests using one technique over the other. Therefore, using the Bartee and Bio-Col alveolar preservation technique is suggested in combination with a xenograft in uniradicular sites.

One of the limitations of this study was the elevation flap, designed to preserve as much keratinized gingiva as possible. The second limitation was the presence or absence of contiguous teeth to the preserved site.

## Figures and Tables

**Table 1:** General characteristics of the patients and alveolar ridge preservation site included in the study.

Patient	Age	Gender	Dental record	Alveolar ridge preservation technique	Implant placement site
1.	62	Male	1.3	Bartee	1.3
			2.3	Bartee	2.3
			3.1	Bartee	—
			1.1	Bio-Col	—
			2.1	Bio-Col	—
			2.2	Bio-Col	—

2.	63	Male	1.3	Bartee	1.3
			2.1	Bartee	2.1
			2.5	Bartee	2.5
			2.3	Bio-Col	2.3
			2.4	Bio-Col	2.4

3.	61	Female	1.1	Bartee	1.1

4.	58	Male	1.2	Bio-Col	—
			1.1	Bio-Col	—

**Table 2:** The height and width resorption percentage by alveolar ridge preservation technique at 6 months of healing.

Variable	Bartee technique		Bio-Col technique		*⁣* ^*∗*^ *p* Value
	Mean	Standard deviation	Mean	Standard deviation	
Resorption in height (%)	11.48	9.82	13.24	7.75	0.482
Resorption in width (%)	21.95	7.46	20.55	7.38	0.949

*⁣* ^*∗*^Wilcoxon signed-rank test.

**Table 3:** Percentage of new bone and residual particles by alveolar preservation technique at 6 months of healing.

Variable	Bartee technique		Bio-Col technique		*⁣* ^*∗*^ *p* Value
	Mean	Standard deviation	Mean	Standard deviation	
New bone (%)	31.10	3.81	30.95	20.36	0.744
Residual particles (%)	28.09	13.56	26.83	27.69	0.302

*⁣* ^*∗*^Mann–Whitney *U* test.

## Data Availability

The data used to support the findings of this study are available from the corresponding author upon request due to ethical conditions established in the informed consent.
